# Characterization of Anopheline unique peroxidase and its role in the regulation of Plasmodium development

**DOI:** 10.1186/1475-2875-13-S1-P49

**Published:** 2014-09-22

**Authors:** Mithilesh Kajla, Parik Kakani, Tania Pal Choudhary, Kuldeep Gupta, Rini Dhawan, SK Gakhar, Lalita Gupta, Sanjeev Kumar

**Affiliations:** 1 Molecular Parasitology and Vector Biology Laboratory, Department of Biological Sciences, Birla Institute of Science and Technology, Pilani, Rajasthan, India; 2 Department of Life Sciences, Maharashi Dayanand University, Rohtak, Haryana, India

## Background

Malaria is major health problem in tropical and subtropical countries of the world. The WHO reported, 207 million malaria cases and 627,000 deaths in 2013 [1]. Malaria is caused by Plasmodium, which completes its asexual cycle in human host and sexual cycle in the female Anopheline mosquito. In order to combat malaria several strategies are in progress, blocking Plasmodium development inside mosquitos is one of them. For this transmission blocking approach we need to understand mosquito immune system and its interaction with Plasmodium at molecular levels. In the African malaria vector, *Anopheles gambiae*, a peroxidase HPX15 (IMPer) is reported to modulate mosquito immunity against Plasmodium [2]. Furthermore we are interested in understanding the regulation of Plasmodium development by its orthologous peroxidase-mediated immune responses in major Indian malaria vectors *An. stephensi* and *An. culicifacies.*

## Materials and methods

We took advantage of *An. gambiae* and other insects with known genomic sequences to explore the peroxidases of *An. stephensi* (AsP-15) and *An. culicifacies* (AcP-15). We amplified *An. stephensi* midgut and carcass cDNA with degenerate primers and the resulting 420 bp fragment was cloned, sequenced and blasted to confirm its identity. Using gene specific primers, tissue-specific and developmental-specific expression as well as its immune role against bacteria, fungus and *P. berghei* was studied. Phylogenetic analysis was carried using Mega 5.02. Silencing of AsP-15 in adult females was achieved by dsRNAi to confirm its role in immunity.

## Results

Sequence analysis revealed a peroxidase, similar to HPX15 of *An. gambiae*, so it was named as AsP-15 and AcP-15 in *An. stephensi* and *An. culicifacies*, respectively. AsP-15 is induced in midgut after blood feeding and pupal stage of mosquito. It is inducing against bacterial and fungal elicitors. Phylogenetic analysis reveals that it is a unique peroxidase, which is evolutionary conserved among Anophelines.

## Conclusions

AsP-15 is an ortholog of HPX15 and it may have a role in modulation of *An. stephensi* gut immunity against blood borne elicitors. Phylogenetic analysis reveals that HPX15, AsP-15 and AcP-15 are unique to Anophelines and this may be due to the involvement of this peroxidase in regulation of Plasmodium development.

## References

[B1] WHO report 2013 Geneva: WHO

[B2] Kumar S Molina-Cruz A Gupta L Rodrigues J Barillas-Mury C A peroxidase/dual oxidase nsystem modulates midgut epithelial immunity in *An. gambiae* Science 2010 327 1644 1648 10.1126/science.1184008 20223948 PMC3510679

